# Management inputs, site conditions, and fire history shape outcomes of invasive plant control and native recovery

**DOI:** 10.1002/eap.70187

**Published:** 2026-02-17

**Authors:** Justin M. Valliere, Olivia A. Parra, Joseph Algiers

**Affiliations:** ^1^ Department of Plant Sciences University of California Davis Davis California USA; ^2^ Santa Monica Mountains National Recreation Area Calabasas California USA

**Keywords:** adaptive management, herbicide application, monitoring, native plant restoration, nonchemical weed control, weed management

## Abstract

Millions of dollars and countless hours are spent on invasive plant management, and the field of invasion ecology has gained increasing attention in recent decades. Yet, despite these efforts to control and understand plant invasions, successful management is often elusive. Budgetary constraints are a common factor limiting invasive plant management programs, and therefore optimizing control strategies is essential. However, such optimization requires data on management inputs and outcomes, and these data are often missing, lacking, or underutilized. To address this knowledge gap and identify predictors of successful invasive plant control in natural areas, we examined nearly 20 years of invasive plant treatment data in the world's largest urban national park—Santa Monica Mountains National Recreation Area of southern California. We resurveyed 279 sites, which had undergone control in the past two decades, collecting data on the abundance of native and invasive plant species to evaluate long‐term management outcomes. We used multiple statistical approaches to identify management inputs and site characteristics that are predictors of eradication, invasive plant cover, and native species recovery. We found that the greater the initial size or percent cover of an infestation, the lower the probability of eradication. We also found that infestations on steeper slopes and in areas that have burned more frequently are less likely to be eradicated. Promisingly, our results also showed that greater reductions in invasives generally benefited native plant communities, though not in all cases. These analyses also highlighted that persistence is key; more frequent treatments (both chemical and nonchemical) and greater investment of labor resulted in larger reductions in invasive plants. Our results highlight how site characteristics and limited resources can complicate invasive plant management, while demonstrating the value of analyzing treatment and monitoring data to identify effective control strategies and guide adaptive management decisions.

## INTRODUCTION

Invasive plant control is a critical component of land stewardship (Foxcroft et al., 2013; Hulme et al., 2014). As such, millions of dollars and countless hours are spent on invasive plant management (Pimentel et al., 2005), and the field of invasion ecology has gained increasing attention in recent decades (James et al., 2010). Yet, despite massive efforts to control and understand plant invasions, successful management is challenging, failure is common, and we still have much to learn to meet the needs of practitioners (Funk et al., 2020; Weidlich et al., 2020). Furthermore, there is still a lack of empirical data on the outcomes of control efforts. Much of the existing literature is based on small‐scale studies conducted over short timeframes (Abella, 2014; Kettenring & Adams, 2011). These species‐specific control trials are unquestionably valuable for developing effective management strategies, and there are an overwhelming number of species which are understudied in this regard (Abella, 2014). However, such trials fail to capture the complexity and larger temporal and spatial scales involved in real‐world land management practices. This may in part explain why many practitioners find peer‐reviewed literature on invasive plant control to be of limited practical use (Matzek et al., 2014).

The key to assessing the effectiveness of invasive plant management programs lies in robust monitoring data, especially from long‐term monitoring, paired with detailed records of management inputs (Blossey, 1999; Dewey & Andersen, 2004; Maxwell et al., 2009). Analyzing these data is essential for guiding adaptive management, allowing managers to optimize control strategies based on what is or is not working. This also enables assessment of the costs and resources required for treatment approaches, which are essential for budgeting and justifying expenses associated with invasive plant control, though this is rarely evaluated (Abella, 2014; Kettenring & Adams, 2011). This is especially true in large protected areas, such as national parks, where managers are tasked with combating numerous invasive species and populations simultaneously, often with limited budgets and personnel (Beaury, Fusco, et al., 2020). Collecting and analyzing such data, however, remains a considerable challenge. The impact of invasive plant control on the recovery of native plant communities is also rarely assessed, with most evaluations of management outcomes narrowly focused on whether or not the target species was eradicated or suppressed (García‐Díaz et al., 2020; Kettenring & Adams, 2011; Reid et al., 2009).

Invasive plant species (i.e., nonnative species that are introduced due to human activities, spread, and cause ecological, environmental, or economic harm) are a well‐recognized threat to the biodiversity, provisioning of ecosystem services, and cultural resources within lands protected by the United States National Park Service (Allen et al., 2009; Stohlgren et al., 2013). As such, considerable resources are invested in invasive plant control within the National Park system, with documented successes. For example, a review of 56 studies evaluating invasive plant treatments across 35 National Parks found that 87% of the studies reported a reduction in targeted invasive species following at least one treatment (Abella, 2014). While this finding is promising and demonstrates that park programs have identified effective treatments for focal species, as seen in other syntheses (Kettenring & Adams, 2011), most studies were limited by short durations and small, site‐specific scales, restricting the understanding of long‐term and landscape‐level outcomes. Furthermore, despite evidence of successful control efforts, invasive plant species are pervasive and increasing within parks (Miller et al., 2021; Stohlgren et al., 2013). Comprehensive evaluations of management efforts over time, spanning multiple sites and species within a given park, could yield valuable insights to better inform how limited resources are allocated.

Adopting a landscape‐level, long‐term approach, we sought to identify the key drivers influencing invasive plant management outcomes in Santa Monica Mountains National Recreation Area (SMMNRA)—the world's largest urban national park. Situated in the greater Los Angeles area, the park spans over 60,000 ha, encompassing a diversity of ecosystems. As with much of California, SMMNRA has experienced significant ecological impacts from invasive plants since the initial wave of introductions during European colonization (Mooney et al., 1986). Today, the park harbors over 300 documented nonnative (i.e., introduced) species, including numerous problematic invasives (Althoen et al., 2007). Invasive plant management within the park is also complicated by environmental change, including increased frequency of large wildfires (Stork et al., 2023), impacts of severe drought on native vegetation (Valliere et al., 2017), and atmospheric nitrogen deposition due to air pollution (Valliere et al., 2020; Valliere et al., 2024), all of which may facilitate nonnative plant invasion.

Invasive plant control is a major management priority within the park, aimed at protecting and restoring native ecosystems, with substantial time and resources dedicated to these efforts. To evaluate the effectiveness of these management activities, we analyzed two decades of control data and conducted resurveys across 279 sites with diverse treatment histories. The resulting dataset encompasses nearly 140 hectares of managed infestations and over 7000 person‐hours of labor. Our objectives were to (1) identify site‐level factors (e.g., slope, aspect, and fire history) shaping management outcomes; (2) examine the impact of management inputs, including labor hours, treatment frequency, and control methods, on achieving successful control of target invasives; and (3) evaluate how these efforts affect the recovery of native plant communities. This comprehensive approach will not only enhance our understanding of invasive plant management within complex, real‐world settings but also offer valuable guidance for optimizing resource allocation and improving restoration outcomes in protected areas worldwide.

## METHODS

### Study site

SMMNRA (34°06′14″ N 118°36′09″ W) is a 63,800‐ha protected area in the Santa Monica Mountains of southern California, northwest of Los Angeles. The park consists of a collection of federal, state, and other public and private lands and is overseen by the US National Park Service. Vegetation consists of a mosaic of coastal sage scrub, chaparral, oak woodlands, riparian woodlands, and grasslands across the landscape, which are directly adjacent to dense urban and suburban areas. The region experiences a Mediterranean climate, with hot dry summers and cool, mild winters during which the majority of rainfall occurs. Mean annual precipitation for the park is approximately 400 mm and shows a high degree variability. Native plant communities have been heavily invaded by a variety of invasive plant species (i.e., introduced plant species that are not native to the region and cause measurable ecological, environmental, or economic harm).

### Invasive plant management and monitoring

Controlling invasive plants within the park is accomplished through a variety of chemical (i.e., herbicides) and nonchemical means (e.g., manual pulling, mowing, and brush cutting). Park staff collect and maintain detailed information on control efforts. When an infestation is selected for control, it is assigned a unique identifier and data on the location, size, and percent cover of the target invader is collected. During each treatment, staff record the methods implemented and labor hours invested. Priority species for control include *Carduus pycnocephalus* (Italian thistle), *Centaurea solstitialis* (yellow starthistle), *Conium maculatum* (poison hemlock), *Euphorbia terracina* (carnation spurge), *Foeniculum vulgare* (fennel), *Lepidium latifolium* (perennial pepperweed), *Phalaris aquatica* (Harding grass), and *Spartium junceum* (Spanish broom).

We made use of nearly 20 years of invasive plant monitoring and treatment data from 279 infestations within the park. We compiled data on treatment management inputs (i.e., treatment methods, treatment frequency, and labor hours) and site characteristics derived from ArcGIS including slope, elevation, and fire history (i.e., fire frequency, fire return interval, and years since last fire). Infestations spanned 11 different park units within the NRA, and included multiple vegetation types, mostly grasslands (44%) as well as urban open space (18%), upland woodlands (16%), riparian woodlands (14%), and chaparral and coastal sage scrub (6%). We calculated the number of times an infestation was treated by method type, including foliar spot spraying of herbicide (i.e., targeted application of herbicide directly onto individual plants or small patches, such as with a backpack sprayer), foliar broadcast of herbicide (i.e., uniform application of herbicide over an entire area, often with boom sprayers), mowing, brush cutting or weed whacking, manual removal, cutting and applying herbicide to plant stems, and application herbicide to the basal bark of plants. If the same treatment type spanned days within a one‐month period, we considered this a single treatment. We also recorded if an infestation was treated with chemical or nonchemical methods or a combination of both. The labor hours invested in treating each infestation were summed across treatments and we also calculated effort on a per‐area basis.

In spring 2023, we resurveyed each of these 279 infestations to assess management outcomes. At each site, we collected data on percent cover of the target invasive, native cover, nonnative cover (i.e., nonnatives other than invasive targeted for control), and native richness. These data were collected within the entire polygon outlining the original infestation using site maps as a guide, as this is the site‐level approach treatment crews use when surveying infestations being treated.

### Statistical analysis

We used a combination of parametric tests (e.g., regression, analysis of variance, and chi‐squared tests), nonparametric tests (e.g., Wilcoxon signed‐rank tests), mixed‐effects models, and random forest models to evaluate the outcomes of invasive plant management. All statistical analysis was performed using R version 4.4.1 (R Core Team, 2024). We modeled species separately or pooled them, depending on the goal of each analysis. To evaluate the influence of site‐level conditions and general management inputs, we pooled data across species to identify broad patterns relevant to landscape‐scale decision‐making. The same approach was used for assessing native plant recovery. In these analyses, we examined the influence of the abundance of the invasive species targeted for control at each site, as well as the abundance and richness of other nonnative plant species, on native plant dynamics. We ran separate models for each invasive plant species when evaluating the effects of specific practices (e.g., mowing, brush cutting, and herbicide) because species differ in traits such as growth form, phenology, and life history, which can strongly influence treatments and outcomes.

### Effectiveness of control efforts

We evaluated how management efforts influenced changes in infestation size and percent cover of the five most commonly targeted invasive plant species, including *Carduus pycnocephalus, Centaurea solstitialis, Euphorbia terracina, Lepidium latifolium*, and *Phalaris aquatica* (hereafter referred to by genus). We used pairwise Wilcoxon signed‐rank tests to compare these metrics between the initial site surveys when control began and the most recent monitoring data from 2023 across all sites. We chose this test because data were not normally distributed. We illustrated these changes using density plots. We also calculated the proportion of successfully eradicated infestations.

### Site‐level predictors of eradication success

We used logistic regression to identify site characteristics that were significant predictors of successful eradication, including initial size and cover of the infestation, slope, and fire history. We used generalized linear models with a binomial distribution to evaluate the relationships between these predictors and the probability of eradication across all sites (*n* = 279) and species (*n* = 17). We also explored models in which time since last treatment was included as a covariate.

### Influence of management inputs on control efforts

We explored relationships between management inputs (i.e., times treated, years treated, and labor hours per acre) and outcomes (i.e., change in infestation cover and size) across all species. We explored different regression models for each relationship (e.g., linear, polynomial, and logarithmic) and selected the best fit based on Akaike information criterion (AIC) comparisons. We also explored models with time since last treatment included as a covariate. We used Spearman's rank correlation tests to assess relationships between site characteristics (e.g., slope, fire frequency, initial cover, and infestation size) and management effort metrics, including number of treatments, total labor hours, and labor hours per acre.

### Comparisons of treatment types

We used linear mixed‐effects models to compare the effect of different treatment types (i.e., chemical, non‐chemical, and combined) on the change in infestation size and percent cover of target invasives. In these models, we included the number of times a site was treated as a random effect, since infestations treated with both chemical and nonchemical means were generally treated more times than those that received a single treatment type.

### Using machine learning to identify predictors of control success

We employed random forest regression models to identify the primary factors driving management success for the two most frequently targeted invasive plant species in the park, *Carduus* (*n* = 60) and *Centaurea* (*n* = 75). These were chosen due to their prominence as control priorities and the robust datasets available, with the highest replication of infestation records among all targeted species. Random forest models are well suited for this analysis because they can handle complex, nonlinear relationships, accommodate interactions between predictors, and provide insights even with imbalanced or noisy data, making them ideal for understanding diverse factors influencing management success (Cutler et al., 2007; Prasad et al., 2006).

For each species, we ran separate models for three metrics of control success: (1) change in infestation size; (2) change in percent cover; and (3) whether or not the infestation had been eradicated. We included a broad range of potential predictors in these models, encompassing site‐level characteristics (e.g., initial cover and size of infestations, elevation, aspect, slope, vegetation type, and fire history) and management inputs (e.g., treatment methods, number of treatments, total labor hours, labor hours per hectare, duration of treatment, and time since the last treatment). Additionally, we evaluated whether other management activities were conducted at the site, such as active restoration of native plant species or inclusion in a fuel modification zone where spring mowing is used to reduce fuel loads. Models were constructed using the *rfPermute* (version 2.5.2) package in R, which extends the *randomForest* framework by providing permutation‐based significance tests for variable importance (Archer, 2016; Breiman et al., 2018). These random forest models employ an ensemble machine learning approach, constructing multiple decision trees from different subsets of data and predictors, and aggregating their outputs to improve predictive accuracy while offering insights into the relative importance of each variable. We initially ran exploratory models with the full suite of predictor variables. After ranking all candidate predictors by permutation mean square error (MSE) importance, we refit a random forest on the top eight predictors for *Centaurea* and top six for *Carduus* given the sample sizes for each species (*n* = 75 and *n* = 60, respectively). After identifying the final predictor variables with the greatest explanatory power, we employed a variety of bivariate analyses (e.g., regression and ANOVA) to further validate and illustrate the influence of these variables on management outcomes.

### Outcomes for native plant communities

We applied the same random forest approach to investigate how management activities and site characteristics influenced outcomes for native plant communities. Models were constructed similarly to those described above, using the same set of predictors. Separate models were used to evaluate native plant cover, total native richness, and native species per area. For these analyses, all 279 infestations across all species were included as replicates. We further examined the influence of key predictors on these metrics using simpler statistical methods, such as regression (based on Pearson correlation) and ANOVA, to better highlight their effects.

## RESULTS

### Effectiveness of control efforts

**FIGURE 1:**
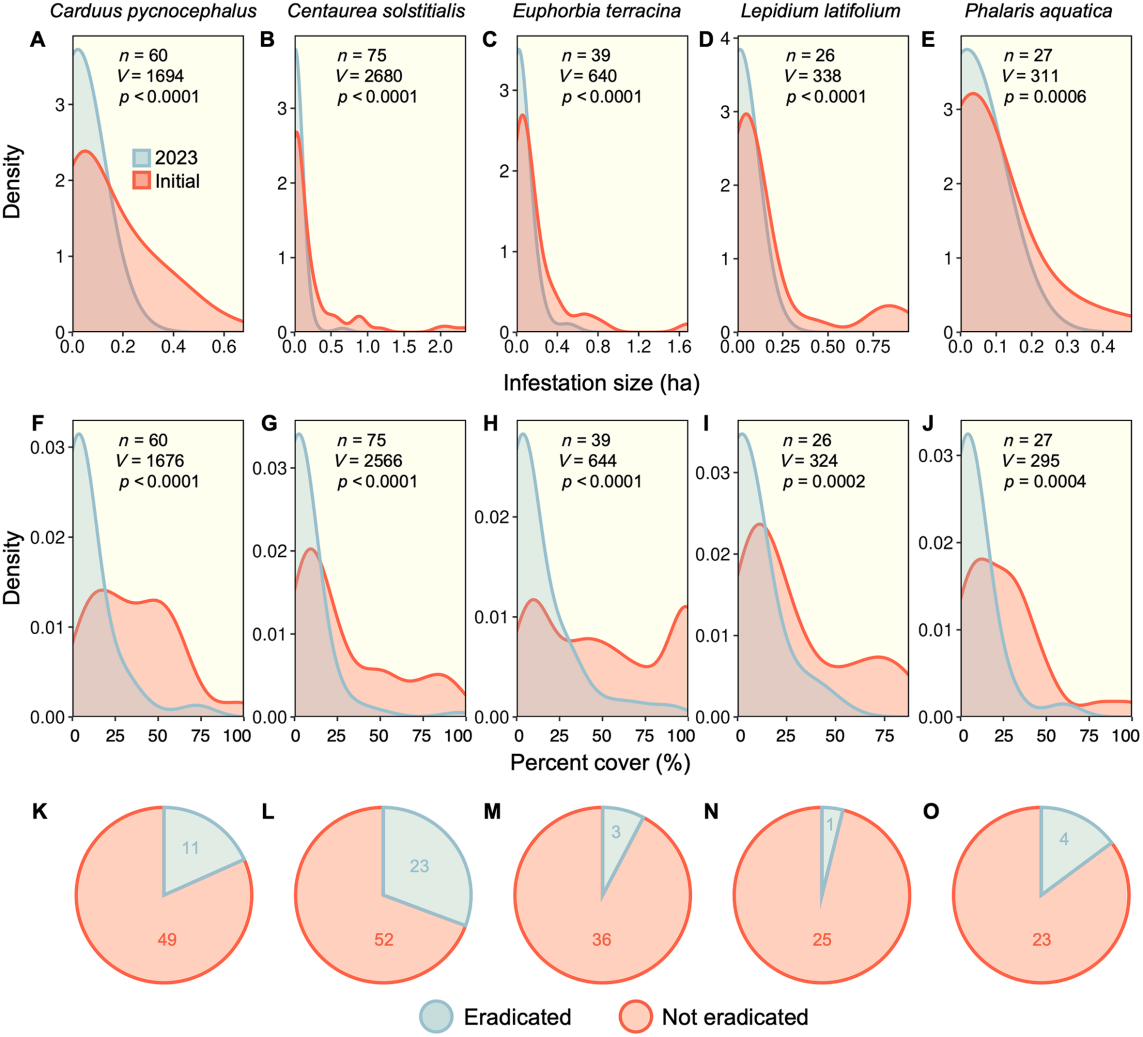
Density plots showing the distribution of sites in terms of infestation size (A–E) and percent cover of the target invasive (F–J) at the first initial treatment and the time of resurveying sites in 2023 for the five most commonly targeted invasive plant species. Results of paired Wilcoxon signed‐rank tests are shown along with sample sizes for each species. Also shown are pie charts representing the proportion of infestations that were successfully eradicated (K‐O).

For the most commonly targeted species, control efforts largely yielded effective reductions in infestation size (Figure 1A–E) and percent cover (Figure 1F–J). Despite these positive shifts, relatively few were fully eradicated (Figure 1K–O). Overall, eradication success was greatest for *Centaurea* and lowest for *Lepidium* and *Euphorbia*. Of the 279 invasive plant infestations evaluated (across all species), 23% were eradicated. Across sites, about 86% showed a reduction in invasive cover and extent, 11% increased, and 3% remained the same.

### Site‐level predictors of eradication success

**FIGURE 2:**
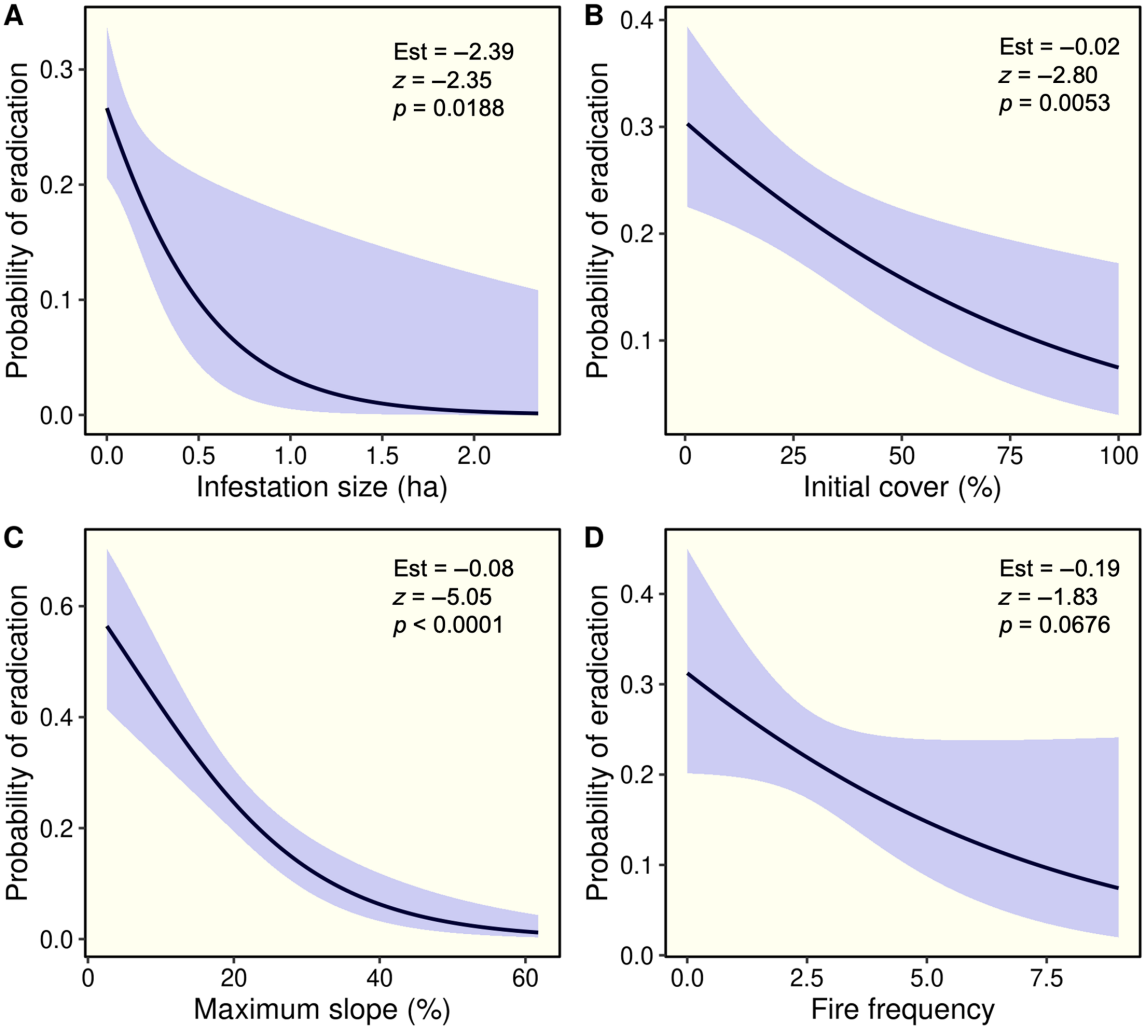
Results of logistic regression models evaluating the influence of site‐level characteristics on the probability of invasive plant eradication, including the initial size (A) and cover (B) of infestations, the maximum slope (C) and fire frequency (D) of sites treated. Predicted regression lines along with 95% confidence bands are shown.

Probability of eradication decreased with increasing infestation size (Figure 2A) and cover (Figure 2B). There was a dramatic reduction in the probability of eradication for infestations on steeper slopes (Figure 2C). We also detected a negative relationship between eradication success and fire frequency (Figure 2D). Likelihood of eradication increased slightly with time since last treatment, with sites with longer periods since the last treatment more likely to have achieved eradication (*z*_1,277_ = 3.37, *p* < 0.001), driven by older sites where eradication success had been achieved and management ceased. In models that included time since last treatment as a covariate alongside site‐level predictors, these variables remained significant, with time since treatment showing a consistent but relatively modest effect (Appendix S1: Table S1).

### Effects of management inputs on invasive plant control

Across species, there was no difference in management outcomes between chemical, non‐chemical, and combined treatment types; changes in percent cover of target invasives (*F*_2,268_ = 0.31, *p* = 0.736) and infestation sizes (*F*_2,268_ = 2.75, *p* = 0.066) were similar across all treatment types. For each of the most commonly targeted species, chemical, nonchemical, and combined treatments yielded similar outcomes (*p* > 0.05 for all models) except for *Lepidium*; sites that received only chemical treatments showed slightly greater control in terms of infestation size compared to sites that received both chemical and nonchemical treatments (*F*_2,21_ = 17.38, *p* < 0.001), but this had no influence on changes in cover (*F*_2,23_ = 0.18, *p* = 0.673).

**FIGURE 3:**
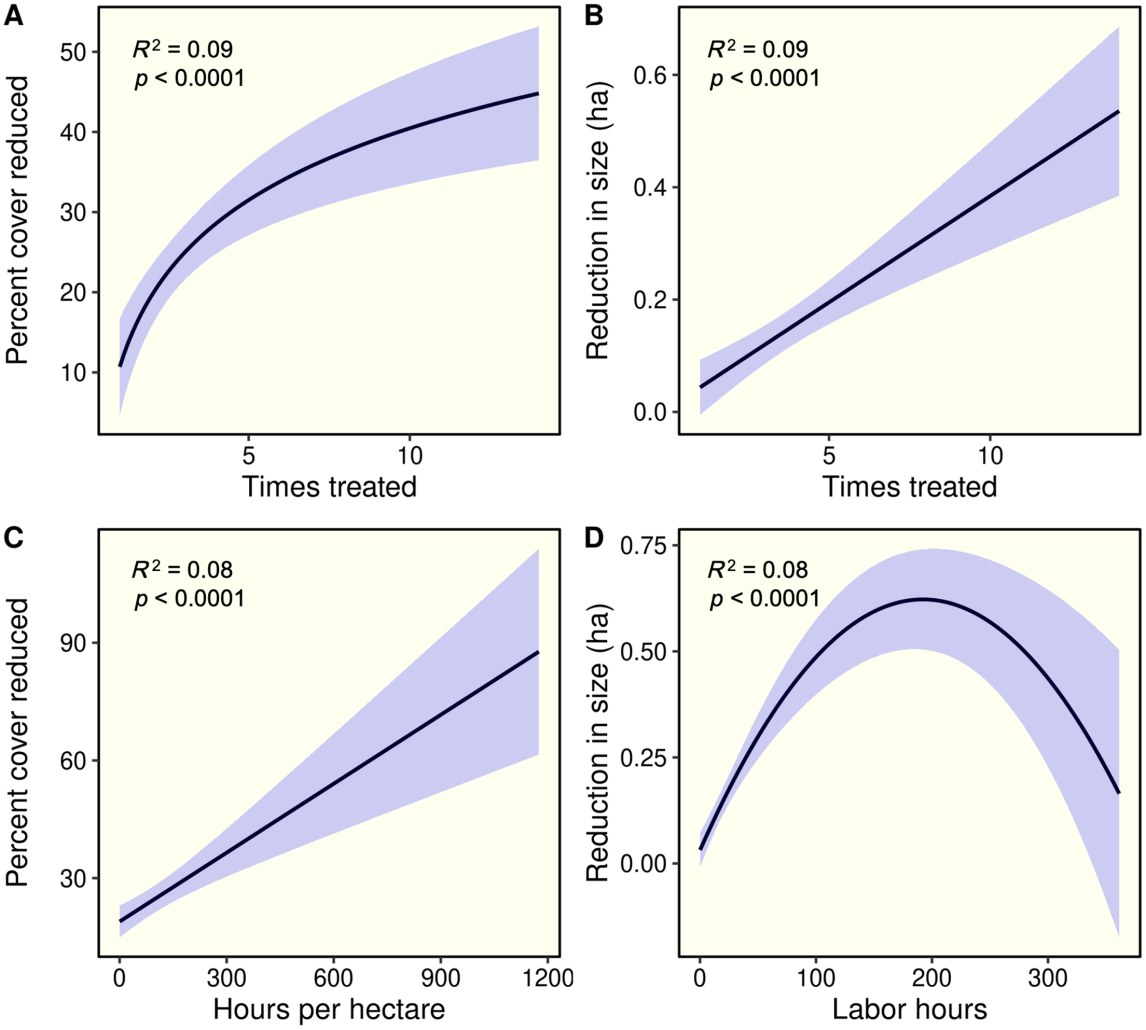
Results of regression models exploring the relationship between the number of times a site was treated and reductions in percent cover (A) and size (B) of infestations, as well as the influence of the number of hours per hectare invested in control and the percent cover reduced (C) and the total number of labor hours invested at a site and the change in infestation size (D). Predicted regression lines along with 95% confidence bands are shown.

There was a positive relationship between the number of times a site was treated and the reductions in percent cover, which was best explained by a logarithmic regression model (Figure 3A). There was a positive linear relationship between the number of times a site was treated and reductions in infestation size (Figure 3B). We detected a strong positive linear relationship between hours per hectare invested in control and percent cover reduced (Figure 3C). We found no relationship between hours per hectare invested in control and reductions in infestation size. However, a nonlinear pattern emerged when examining the total number of labor hours: initial increases in time spent at a site were associated with greater reductions in infestation size, but this effect diminished at sites with the highest time investments (Figure 3D). In models in which time since last treatment was included as a covariate, each of these predictors remained highly significant (Appendix S1: Table S2). Overall, reductions in cover decreased significantly with increasing time since last treatment (*R* ^2^ = 0.04, *p* < 0.001). Longer times since last treatment also showed a positive relationship with infestation size (*R* ^2^ = 0.10, *p* < 0.001).

### Relationships among site conditions and management effort

Spearman correlations revealed several significant relationships between site characteristics and treatment effort across all sites (*n* = 279). Steeper slopes were associated with slightly greater management effort, as indicated by a weak but significant positive correlation with the number of treatments (ρ = 0.15, *p* = 0.011) and a moderate positive correlation with total labor hours (ρ = 0.27, *p* < 0.001). However, slope was not significantly related to labor hours per acre (ρ = −0.05, *p* = 0.391). Fire frequency was not associated with the number of treatments (ρ = 0.01, *p* = 0.924), but it was positively correlated with both total labor hours (ρ = 0.26, *p* < 0.001) and labor per acre (ρ = 0.16, *p* = 0.006). Peak initial cover showed consistent positive correlations with management effort, including number of treatments (ρ = 0.24, *p* < 0.001), total labor hours (ρ = 0.39, *p* < 0.001), and labor per acre (ρ = 0.24, *p* < 0.001). Infestation size was strongly correlated with both the number of treatments (ρ = 0.44, *p* < 0.001) and total labor hours (ρ = 0.66, *p* < 0.001), but showed no significant relationship with labor hours per acre (ρ = −0.04, *p* = 0.509).

### Predictors of management success: *Centaurea solstitialis*

**FIGURE 4:**
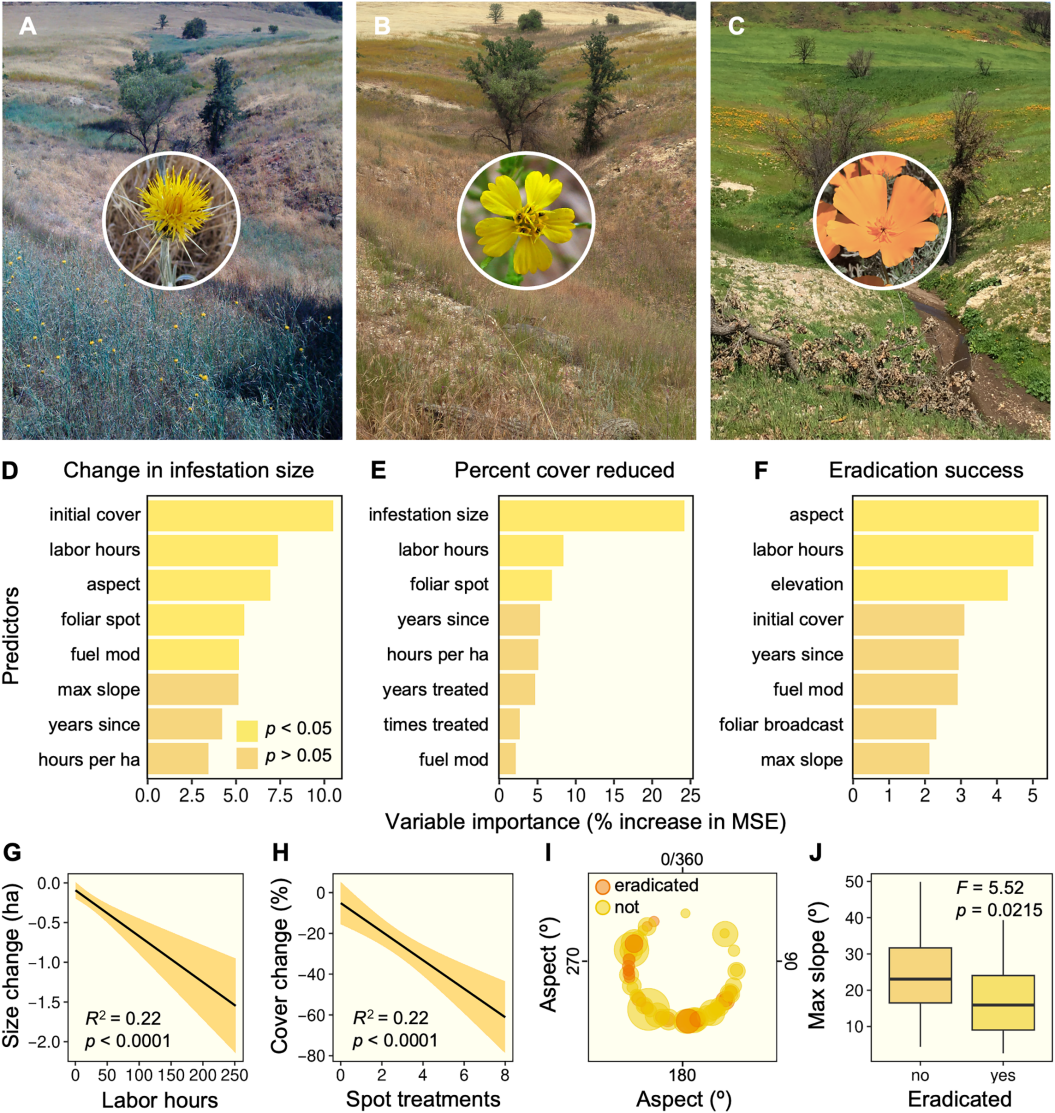
Potential drivers of successful management for *Centaurea solstitialis* infestations (A). In some sites where successful control has been achieved, native wildflowers such as *Deinandra fasciculata* (B) and *Eschscholzia californica* have returned (C). Significant predictors from random forest models are displayed for changes in infestation size (D), reductions in cover (E), and eradication success (F). Significant predictors are shown in yellow. Bivariate analyses highlight key factors influencing management outcomes, including the effect of labor hours on infestation size (G), the number of herbicide spot treatments on changes in cover (H), the influence of slope aspect and infestation size (indicated by circle size) on eradication success (I), and the influence of slope on eradication success (J). Landscape photographs (A–C) by Joseph Algiers, National Park Service; inset photographs of flowers by Anthony Valois.

*Centaurea* was the most commonly targeted species (*n* = 75). Reductions in infestation size (Figure 4D) were best explained by initial cover (12%), number of hours spent on control (11%), slope aspect (8%), number of foliar spot treatments applied (7%), and whether or not the infestation was located in a fuel modification zone (7%). Reductions in invasive cover (Figure 4E) were best explained by initial infestation size (24%), labor hours invested (11%), and number of foliar spot treatments applied (8%). Eradication success (Figure 4F) was best explained by slope aspect (5%), labor hours invested in control (5%), and elevation (4%). Increased investment in labor hours for treatment resulted in greater control success in terms of infestation size (Figure 4G), and greater reductions in *Centaurea* cover were achieved at sites with more frequent spot treatments of herbicide (Figure 4H). Slope aspect also appeared to influence management outcomes (Figure 4I), where smaller infestations on west‐facing slopes were more likely to be eradicated than those on south‐facing slopes. Infestations on steep slopes were more difficult to eradicate (Figure 4J).

### Predictors of management success: *Carduus pycnocephalus*

**FIGURE 5:**
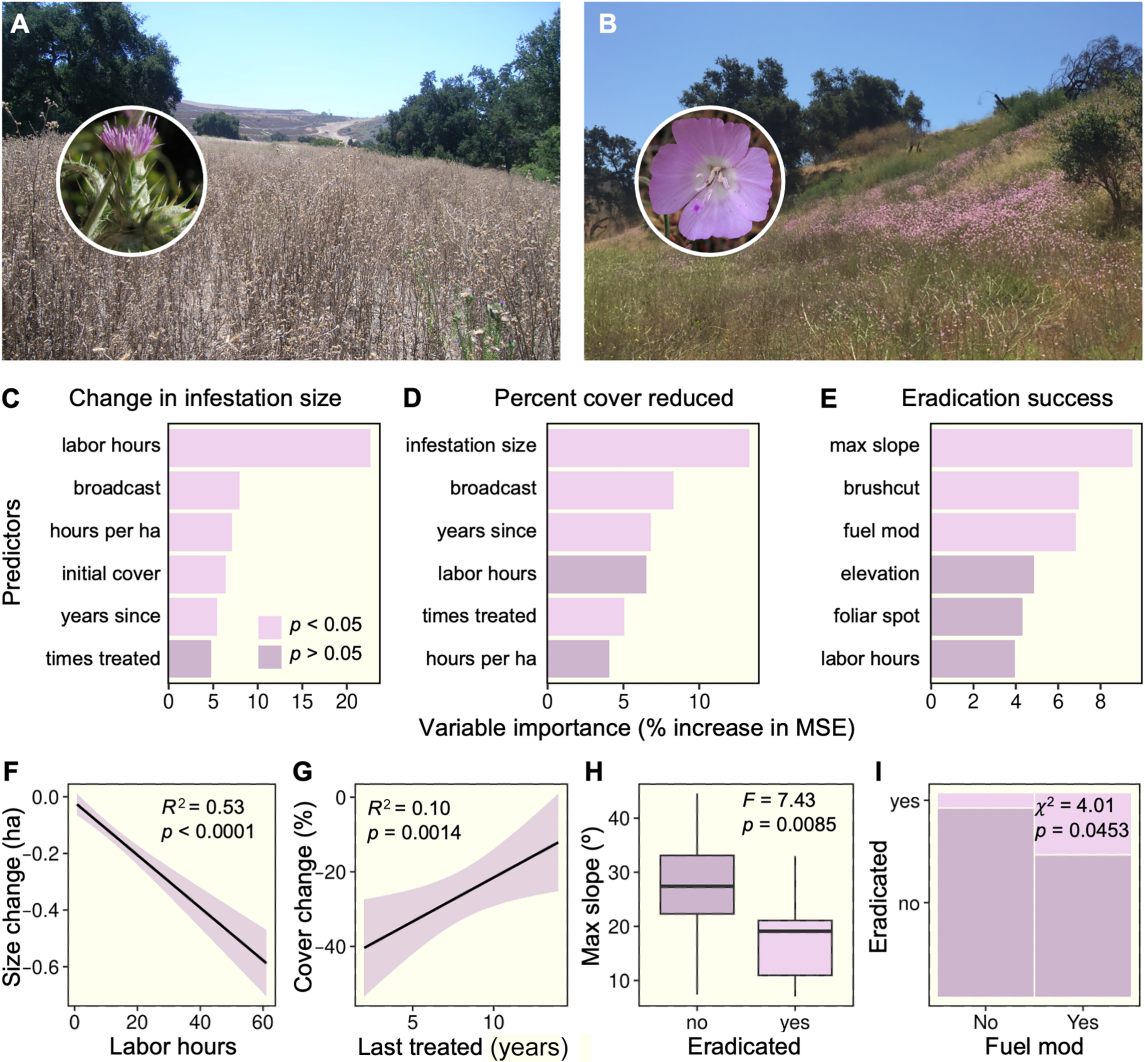
Predictors of successful management for *Carduus pycnocephalus* infestations (A). In some sites where successful control has been achieved, native wildflowers such as *Clarkia* species have returned (B). Significant predictors from random forest models are displayed for changes in infestation size (C), reductions in cover (D), and eradication success (E). Significant predictors are shown in light purple. Bivariate analyses highlight key factors influencing management outcomes, including the effect of labor hours on infestation size (F), time since last treatment on percent cover (G), the influence of slope on eradication (H), and the proportion of eradicated infestations inside or outside fuel modification zones (I). Landscape photographs (A–B) by Joseph Algiers, National Park Service; inset photographs of flowers by Anthony Valois.

*Carduus* was the second most commonly targeted species (*n* = 60). Changes in infestation size (Figure 5C) were best explained by number of hours invested (24%), number of broadcast herbicide applications (8%), hours per hectare invested (7%), initial cover (7%), and years since last treatment (4%). Significant predictors of reductions in cover (Figure 5D) included initial infestation size (16%), number of broadcast herbicide applications (11%), years since the last treatment (10%), and total number of times a site was treated (6%). Eradication success (Figure 5E) was best explained by maximum slope of sites (10%), number of times a site was treated with brush cutters (7%), and whether or not a site was located in a fuel modification zone (7%). The greater the labor hours invested in control, the larger the reductions in infestation size (Figure 5F). There was a positive relationship between the number of years since a site was last treated and the change in percent cover (Figure 5G). Eradication success was significantly lower for sites on steeper slopes (Figure 5H) and more likely to be achieved in fuel modification zones (Figure 5I).

### Outcomes for native plant communities

**FIGURE 6:**
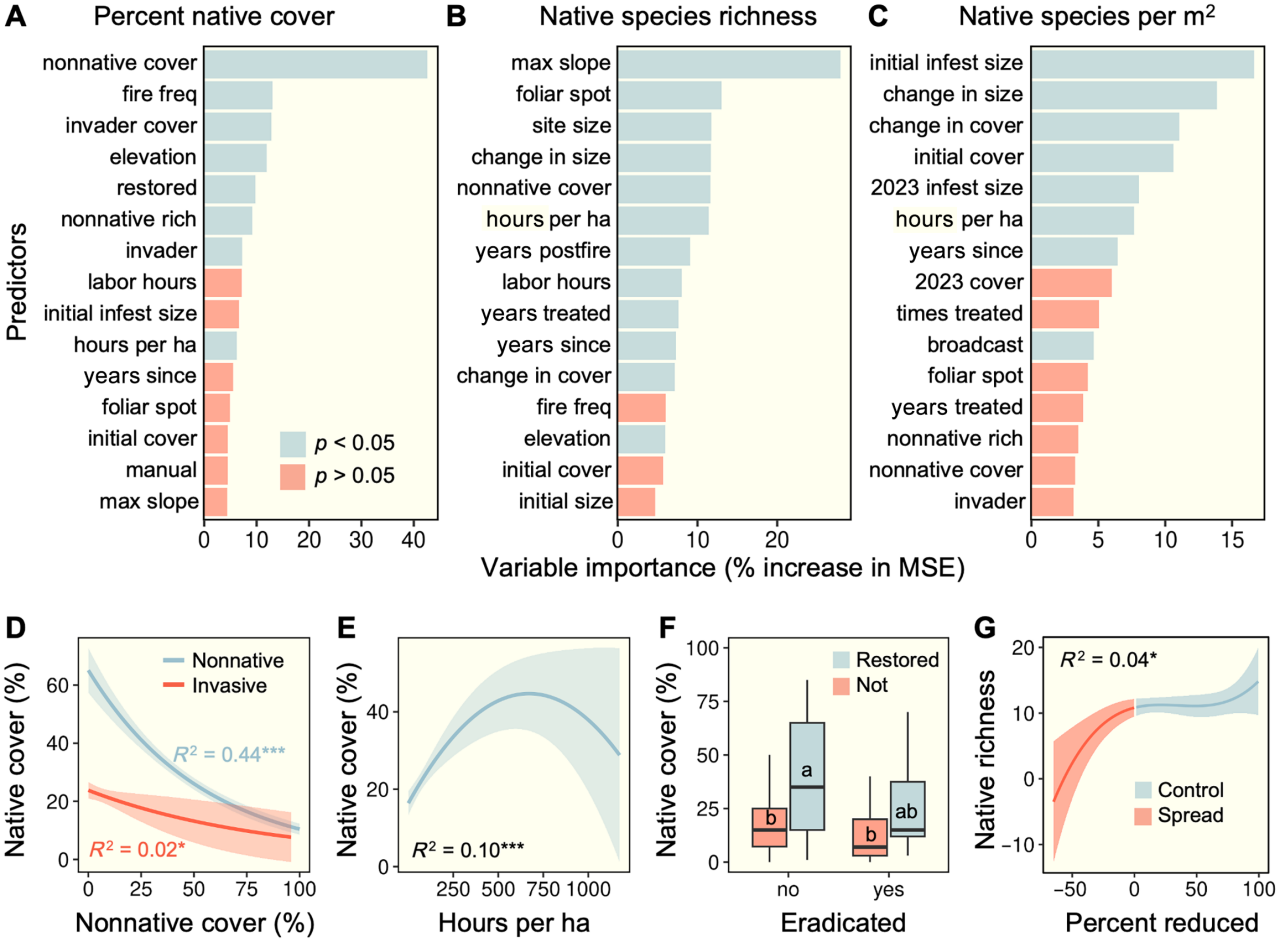
Results of random forest models assessing the influence of site‐level variables and management inputs on percent native cover (A), native richness (B), and the number of native species per area (C). Significant predictors are shown in blue. Key relationships between significant predictors and native plant communities are also illustrated: regressions showing the relationship between nonnative plant cover (both total nonnative cover and that of just targeted invasive species) and native cover (D); the relationship between labor hours per hectare and native cover (E); boxplots comparing native plant cover in eradicated versus non‐eradicated sites, including both restored and unrestored areas (F); and the effect of the percent reduction in target invasive cover during the treatment period on native plant richness across all sites (G). Each plot's legend shows what colors represent in that specific graph.

Random forest models identified multiple site characteristics and management inputs that influenced native plant community responses to invasive plant control (Figure 6). The top predictors of native plant cover (Figure 6A) were total nonnative cover (43%), fire frequency (13%), invasive plant cover (13%), elevation (12%), restoration status (10%), nonnative richness (9%), target invasive identity (7%), and hours per hectare invested in control (7%). Native richness (Figure 6B) was best predicted by maximum slope of the site (27%), the number of foliar spot treatments applied (14%), site size (12%), change in infestation size (11%), nonnative cover (11%), hours per hectare invested (10%), years since the last fire (9%), the total labor hours invested (7%), number of years treated (7%), time since the site was last treated (7%), change in invasive cover (6%), and elevation (5%). The number of native plant species per square meter was best explained by initial infestation size (17%), change in infestation size (14%) and cover (11%), invasive cover at the time of first treatment (11%), infestation size in 2023 (8%), years since last treatment (6%), and frequency of broadcast herbicide treatments (5%).

Native plant cover significantly declined as total nonnative cover increased, while cover of the targeted invasive plant species was associated with even lower native cover (Figure 6D). The relationship between hours per hectare invested in control and native cover was nonlinear; while increased control efforts generally improved native cover, sites with greater labor investment exhibited lower and more variable native cover (Figure 6E). Native cover was slightly lower in sites where the target invasive had been eradicated (*F*_1,275_ = 5.36, *p* = 0.021) and substantially higher in restored sites (*F* = 18.56, *p* < 0.001), but there was no interaction (*F*_1,275_ = 0.06, *p* = 809) between restoration or eradication status (Figure 6F). Native richness did not differ between restored and unrestored sites (*F*_1,275_ = 0.15, *p* = 0.697). Native plant richness was affected by changes in invasive plant cover, though the relationship was not linear (Figure 6G). Sites with substantial increases in invasive cover saw sharp declines in native richness, whereas sites with minimal changes in cover maintained stable richness. The greatest native richness was observed at sites with the most effective control. Native richness increased linearly with the number of years treated (*R* ^2^ = 0.10, *p* < 0.001). Finally, native richness showed a weak but significant negative relationship with invasive cover in 2023 (*R* ^2^ = 0.02, *p* < 0.001). There was no relationship between native richness and invasive cover at the time control was initiated (*R* ^2^ < 0.01, *p* = 0.246).

## DISCUSSION

Invasive plant management is an inherently challenging task (D'Antonio et al., 2004; Pearson & Ortega, 2009). Drawing on a long‐term dataset from a large, urban national park, we show that sustained investment in control generally reduces infestation severity while benefiting native plant communities—an outcome often assumed but rarely documented (Reid et al., 2009). Although complete eradication was uncommon, most (86%) infestations declined in cover and extent, with variability in success explained by differences in site‐level factors. This rate of success is remarkably similar to that reported by Abella (2014) in a study examining many individual control trials across the US National Park System. Our findings underscore both the potential for meaningful progress for land management and the persistent challenges practitioners face, particularly under widespread constraints of limited funding and personnel (Beaury, Fusco, et al., 2020; Bradley et al., 2010). More broadly, they illustrate the value of monitoring data for identifying best practices and barriers to success (Abella, 2014). Such data can help also justify sustained investment to policymakers and maintain management momentum as global change accelerates invasion pressures (Bradley et al., 2010; Early et al., 2016).

### Site conditions and history complicate control efforts

A strength of this study lies in its inclusion of many sites with diverse environmental conditions and management histories. Our findings demonstrate that a variety of site‐level factors significantly influence management outcomes. This knowledge can inform the prioritization and optimization of control efforts. For instance, larger and denser infestations were more challenging to control and had greater negative impacts on native plant communities. This underscores a fundamental principle in invasive plant management: where possible, prioritizing early treatment of new infestations is the most cost‐effective strategy to minimize impacts on native ecosystems (Abella, 2014; DiTomaso et al., 2007). Globally and across ecosystems, increasing fire frequency heightens the risk of nonnative plant invasion (Early et al., 2016), and we show that frequent fires also severely hinder management efforts. Many invasive plant species are disturbance‐adapted and tend to proliferate following fire events (Lambert et al., 2010). More frequent fires may also prevent native perennials from fully regenerating, which increases susceptibility to invasion (Keeley & Brennan, 2012). Prioritizing control efforts and native plant restoration immediately after fires, especially in areas with a history of recent burns, may be a critical strategy for disrupting the invasion–fire cycle. Finally, we found that infestations on steeper slopes were much more difficult to control, which is troubling because these sites often tended to have higher native plant diversity. This is likely because steeper sites are more difficult to access and navigate, which can lead to less thorough treatments. Such sites may need to be given higher priority and treated by well‐trained crews to overcome this challenge—one that is likely universal across ecosystems that occur on uneven or rugged terrain.

### Persistence and diverse strategies drive success

Our results show that persistence pays off; greater effort and more frequent treatments generally improved invasive plant control. Yet, the relationships between effort and outcomes were sometimes nonlinear, with signs of diminishing returns where additional labor did not yield proportional gains. Even more concerning, there were some sites where greater effort was associated with lower success. These sites may represent “treatment traps” that necessitate adopting adaptive management approaches to achieve effective control (Foxcroft & McGeoch, 2011). These may include shifting objectives from eradication to containment, adjusting treatment timing or methods (e.g., integrating multiple techniques or targeting infestations earlier in the growing season), prioritizing higher feasibility sites, or implementing measures to prevent reinvasion from nearby populations. Lastly, premature abandonment of management can allow infestations to persist or spread; longer gaps since the last treatment were linked to lower reductions in density and size, underscoring the need for consistent, timely interventions. These patterns highlight principles that extend beyond our study system: tailoring effort to site conditions, identifying when to change tactics, and maintaining consistent interventions are critical for successful invasive plant management in diverse landscapes. Furthermore, documenting ineffective treatments can be as valuable as recording successes, as these experiences can reveal why certain approaches fail and inform the development of more effective strategies. For instance, in Florida's National Parks, eventual control of multiple aggressive invaders was achieved only after managers adapted methods based on lessons learned from earlier, ineffective treatments (Abella, 2014; Dalrymple et al., 2003).

Employing multiple treatment strategies, both chemical and nonchemical, is often key to success. Foliar broadcast herbicide treatments can efficiently manage large infestations, but our results show that frequent, targeted spot treatments are critical for sustained control, particularly for species like *Centaurea*. We also found that chemical, nonchemical, and mixed approaches achieved similar control for most species, suggesting that nonchemical methods can be effective alternatives where herbicide use is limited. An exception was *Lepidium*, where mechanical control was less successful, consistent with its ability to resprout from root fragments (Young et al., 1998). Overall, control is often most effective when multiple approaches are integrated. For example, control of *Centaurea* and *Carduus* was greatest in fuel modification zones, where annual mowing was combined with herbicide treatments. Such multi‐pronged strategies can maximize impact across a variety of species and site conditions. We did observe some notable species‐specific responses. For example, while both *Carduus* and *Centaurea* showed that infestation severity (e.g., size, cover) and labor investment influence control outcomes and steep slopes hinder success, random forest models revealed species‐specific differences. For *Carduus*, the frequency of broadcast herbicide treatments was a strong predictor of control, consistent with its susceptibility to herbicides, relatively weak competitiveness, and limited seedbank persistence (DiTomaso et al., 2013). In contrast, *Centaurea* is a highly aggressive invader considered among California's most problematic species (Pitcairn et al., 2006). It produces abundant seed and cannot be controlled with a single treatment (DiTomaso & Kyser, 2013), making follow‐up spot treatments critical for success.

### Lack of eradication success does not mean failure

On the surface, low rates of eradication success may seem discouraging, but there are several considerations that may offer reassurance to land managers. First, most infestations in this dataset involve widespread, well‐established invasive species rather than the localized targets of early detection rapid response (EDRR) campaigns (Reaser et al., 2020). For such large, established populations, complete eradication is more challenging (García‐Díaz et al., 2020), often unrealistic, and not necessarily the primary objective (Head et al., 2015). Abella (2014) showed that infestations greater than one hectare were unlikely to be eradicated within National Parks, and our results suggest that this threshold may be even lower. Even without full eradication, management can deliver substantial benefits, including reduced invasive cover, native recovery, and prevention of further spread. This dataset included sites with variable treatment histories, and some sites may not yet be at a stage where management efforts have had sufficient time to yield results, partly due to the site‐specific environmental factors identified. Understanding the complexities of these interactions provides valuable insights for guiding management strategies moving forward (Foxcroft & McGeoch, 2011).

### Does invasive plant control benefit native species?

A key but often overlooked question in evaluating invasive plant control is its impact on native plant communities (Reid et al., 2009). The ultimate goal should not merely be the removal of invasives but rather the restoration and resilience of native ecosystems (García‐Díaz et al., 2020; Pearson & Ortega, 2009). Because native cover and richness data were only collected in 2023, after control began, it is difficult to directly attribute differences in these metrics to management efforts. Yet, several lines of evidence suggest that management does indeed benefit native plant species. First, there was a clear negative effect of invasive plant cover on native richness and cover. Second, reductions in invasive cover were associated with increased native richness, whereas sites where invasives expanded had substantially lower richness. Lastly, increased frequency of treatments and greater investment in labor (up to a certain point) were associated with greater native cover and richness. Collectively, these results align with what one would predict (and hope) with successful management: native species rebound when control efforts are effective. However, invasive plant control alone may not be sufficient for full recovery of native plant communities (Abella, 2014; Reid et al., 2009). For example, sites where invasives were eradicated often had relatively low native cover unless active restoration was implemented. Furthermore, while greater labor investment tended to increase native cover, sites with the greatest hours invested in control had low native cover. This likely reflects variations in site history, disturbance levels, and the availability of native propagules for recolonization. Passive restoration may be sufficient at some sites after control, but others may require active revegetation to support native recovery (DeSimone, 2011; Prach et al., 2020). Reestablishing natives could also help to resist reinvasion (Beaury, Finn, et al., 2020; Levine et al., 2004).

## CONCLUSIONS

Land managers must often make decisions about resource allocation and control strategies without certainty of their effectiveness, relying instead on experience and ecological intuition (DeSimone, 2013). By systematically analyzing past treatment data and resurveying sites, as we have done here, managers can evaluate program success, identify what works (and what does not), and understand why (Abella, 2014). This approach may also help bridge the well‐recognized science–practice gap in invasive plant management (Funk et al., 2020; Weidlich et al., 2020). Although monitoring is frequently constrained by limited resources, it remains an essential component of management (Blossey, 1999). It is possible that investing more time in monitoring could actually improve overall management outcomes even if it comes at the cost of hours spent actively treating infestations (Maxwell et al., 2009). Despite the useful insight offered here, a critical challenge remains identifying the factors that can prevent managers from falling into ineffective treatment cycles, where increased time and effort yield little return. Nonetheless, our findings demonstrate that control efforts can succeed and benefit native plant communities, even if outcomes are not universally successful. While some of our findings may be confirmatory or intuitive, they provide valuable insights into the real‐world dynamics of land management that are absent from carefully controlled experiments. This practical perspective highlights the many challenges practitioners face and the importance of persistence, monitoring, and adaptive management in achieving long‐term success. In an era of accelerating environmental change, invasive plant control remains one of the few interventions that can be directly implemented in protected areas to mitigate impacts, in contrast to largely unmanageable global drivers such as rising temperatures and CO_2_. We hope these results can help refine strategies, inform decision‐making, and ultimately improve outcomes for invasive plant management more broadly.

## AUTHOR CONTRIBUTIONS

Justin M. Valliere and Joseph Algiers conceived the research and designed the sampling methodology. Olivia A. Parra and Joseph Algiers collected the field data. All authors contributed to data organization and management. Justin M. Valliere analyzed the data and wrote the manuscript, with input and review from Olivia A. Parra and Joseph Algiers.

## CONFLICT OF INTEREST STATEMENT

The authors declare no conflicts of interest.

## Supporting information


Appendix S1.

## Data Availability

Data (Valliere et al., 2025) are available in Dryad at https://doi.org/10.5061/dryad.fj6q5747x.
