# Supplementary material for: Management inputs, site conditions, and fire history shape outcomes of invasive plant control and native recovery
Source: Ecol Appl. 2026 Feb 17;36(1):e70187. doi: 10.1002/eap.70187 (PMC12910536; doi:10.1002/eap.70187)
Supplement: Supplementary file 1 — Appendix S1. [file EAP-36-e70187-s001.pdf]

Management inputs, site conditions, and fire history shape outcomes of invasive plant control and native recovery

Justin M. Valliere, Olivia A. Parra, and Joseph Algiers

Ecological Applications

Appendix S1

Table S1. Results of logistic regression models testing the effects of individual site and treatment variables (initial cover, infestation size, slope, and fire frequency) on eradication success, with years since last treatment included as a covariate in each model.

Model	Predictor	Estimate	z-value	p-value
Eradication vs. initial cover	Initial cover	–0.015	–2.44	0.015
	Years since treated	0.127	3.04	0.002
Eradication vs. infestation size	Infestation size	–0.19	–2.43	0.015
	Years since treated	0.09	2.10	0.036
Eradication vs. maximum slope	Max slope	–0.073	–4.61	<0.001
	Years since treated	0.10	2.34	0.019
Eradication vs. fire frequency	Fire frequency	–0.22	–2.08	0.038
	Years since treated	0.14	3.54	<0.001

Table S2. Summary of regression models testing the effects of treatment effort (number of treatments or labor hours) and time since last treatment as a covariate on invasive plant control outcomes.

Model	Predictor	Estimate	F	df	R^2	p-value
Cover reduced vs. times treated	Times treated	12.88	14.25	2, 276	0.094	< 0.001
	Years since treated	–0.015	–	–	–	0.981
Change in size vs. times treated	Times treated	–0.027	16.26	2, 276	0.105	0.003
	Years since treated	0.012	–	–	–	0.043
Cover reduced vs. hours/ha	Hours per acre	0.13	16.65	2, 276	0.108	< 0.001
	Years since treated	–1.44	–	–	–	0.002
Change in size vs. labor hours	Labor hours	–1.64	26.12	3, 275	0.222	< 0.001
	Years since treated	0.009	–	–	–	0.067
